# Coaching Models of School-Based Prevention and Promotion Programmes: A Qualitative Exploration of UK Teachers’ Perceptions

**DOI:** 10.1007/s12310-018-9282-3

**Published:** 2018-07-02

**Authors:** Emma Ashworth, Ola Demkowicz, Ann Lendrum, Kirsty Frearson

**Affiliations:** Institute of Education, University of Manchester, Oxford Road, Manchester, UK

**Keywords:** Coaching, Implementation, School-based interventions, Prevention and promotion, Cultural transferability, Social validity

## Abstract

There has been increased interest in recent years regarding the utility of imported universal prevention and promotion (P&P) programmes in UK schools, many of which have a coaching model attached. However, there have been relatively few studies exploring the cultural transferability and social validity of these models, even though evidence suggests that these factors are important to the successful implementation of the programmes, and thus the achievement of the intended outcomes. The aim of the current study was to explore the coaching practices that teachers report experiencing, and to further understanding of the perceived benefits of these coaching practices to teachers. The sample consisted of 33 teachers implementing one of two universal, school-based P&P programmes, *Good Behavior Game* and *Promoting Alternative Thinking Strategies* as part of large-scale, randomised controlled trials. Qualitative, semi-structured interviews were conducted, and data were analysed thematically utilising a hybrid approach. Teachers typically reported engaging in six distinct practices with their coaches. While the majority of these practices were in line with coaching literature, there were some discrepancies between intended coaching practices and teachers’ reports. The coaching practices were generally perceived to be acceptable to teachers. Two unanticipated practices, *validation* and *motivation*, appeared to be of particular value to teachers, although these are not currently a prominent feature in existing coaching models. The findings provide implications for improving the development of socially valid coaching models for UK schools.

## Introduction

Prevention and promotion (P&P) programmes are interventions that aim to reduce maladaptive behaviours in children before they occur, by developing and enhancing the skills and strategies required to preclude negative outcomes (Lendrum & Wigelsworth, 2013; Stallard, 2013). P&P programmes have a well-established evidence base that demonstrates their success in improving outcomes for children. However, there is evidence to suggest that the way these programmes are implemented can have an impact on their outcomes (Durlak & DuPre, 2008). Therefore, these programmes often have a coaching element designed to support deliverers with implementation, to ensure that the critical components of the programme are implemented correctly, thus increasing the likelihood of successful outcomes. However, while implementation can be influenced by a variety of factors including the programme’s acceptability to the deliverer (Wehby, Maggin, Partin, & Robertson, 2011), little is known about the acceptability of the coaching model, particularly with regard to imported P&P programmes.

P&P programmes were originally implemented in the USA in response to concerns of a generational public health crisis (Hamburg, 1994) and typically targeted at-risk children more likely to experience outcomes such as poor educational attainment, mental health disorders, and school dropout (McLoyd, 1998; Reilly, 2003; Suh & Suh, 2007). However, over time there has been a shift towards a universal approach in delivering P&P programmes within schools, where all pupils are exposed to these programmes, irrespective of risk status (Humphrey, Lendrum, Barlow, Wigelsworth, & Squires, 2013). As well as avoiding the stigma associated with targeted interventions (Poduska et al., 2008), this approach is intended to have an “immunisation” quality, preventing the onset of negative outcomes in the general population through the promotion of adaptive behaviours (Embry, 2004; Lendrum & Wigelsworth, 2013; Merrell & Gueldner, 2010; O’Connell, Boat, & Warner, 2009). These interventions tend to focus on promoting non-academic skills such as social and emotional skills or positive behaviour, with the aim of preventing mental health difficulties and negative behaviours (see World Health Organization, 2006 for an overview).

Many school-based P&P programmes include a coaching model designed to support teachers in the effective implementation of an intervention (e.g. *Promoting Alternative Thinking Strategies* (PATHS), Berry et al., 2015; *Good Behavior Game* (GBG), Coombes, Chan, Allen, & Foxcroft, 2016; *The Incredible Years*, Reinke et al., 2012a, b). Although there has been a growing interest in the potential benefits of coaching (e.g. Pas et al., 2015; Poduska & Kurki, 2014; Reinke et al., 2012a, b; Stormont & Reinke, 2012), there is as yet little consensus on how it should be defined, or what form it should take (Akin, 2016; Nadeem, Gleacher, & Beidas, 2013; Stormont, Reinke, Newcomer, Marchese, & Lewis, 2015). Instead, coaching is often used interchangeably with terms such as “consultation”, “mentoring”, “technical assistance” or “supervision” (Colquhoun et al., 2013; Edmunds, Beidas, & Kendall, 2013; Elliott & Mihalic, 2004; Mannix et al., 2006), which may partly be due to the lack of understanding of the core elements of coaching (Akin, 2016).

Despite these differences, there is general agreement on the distinction between coaching and initial intervention training (Stormont et al., 2015). While the aim of initial intervention training is to equip the implementer with the knowledge and skills to deliver the programme, coaching is seen as an ongoing process that aims to improve the application of knowledge and skills to classrooms (Joyce & Showers, 1981; Stormont et al., 2015). In this approach, coaches may be used to support implementation fidelity, that is, to ensure that the intervention is delivered as intended, so that the mechanisms of change are triggered and the intended outcomes achieved (Dane & Schneider, 1998; Elliott & Mihalic, 2004). Indeed, a review of the research in this area found that engagement with a coach improved the extent to which teachers accurately implemented the programme (Kretlow & Bartholomew, 2010).

There is limited literature regarding the nature of the coaching role and the practices this includes. In an attempt to clarify this, the American Institutes for Research (AIR, 2005) synthesised the literature and identified two distinct but overlapping strands: forms of coaching, and coaching practices. In the AIR model, each form “implies a distinctive purpose of coaching” (AIR, 2005, p. 20) which can vary between contexts and situations (Poglinco, Bach, Hovde, Rosenblum, & Saunders, 2003). Coaching may be *technical*, whereby support is given to implementers in the learning and application of the specific strategies required to attain high levels of fidelity (Joyce & Showers, 1982). Alternatively, it may be *problem solving*, where coaches apply their expert theoretical knowledge of the programme to provide solutions to any issues that teachers are experiencing, ranging from technical issues to participant responsiveness (Joyce & Showers, 1982; Nadeem et al., 2013). Finally, coaching can involve *aiding reflective practice*, whereby teachers are encouraged to think about instances where implementation of the P&P programme was successful, and where improvements could be made to enhance fidelity. All these coaching forms aim to support effective implementation.

Additionally, coaching practices are the activities that coaches engage in with a teacher, although the literature does not suggest specific frequencies or combinations of practices for the different forms of coaching (Hasbrouck & Denton, 2005; Joyce & Showers, 1982). One such practice is *observations*; coaches watch intervention delivery in order to identify teachers’ strengths and weaknesses, and address areas where implementation could be strengthened. Conversely, coaches may engage in *modelling*, delivering a practical demonstration or role-play activity to indicate the core skills needed for effective implementation (Joyce & Showers, 1982). Finally, coaching practices can centre around *communication and feedback*; effective communication is considered to be a cornerstone practice used by coaches to develop successful relationships with teachers (Stormont et al., 2015). Coaches provide information on theory and practice, deliver feedback and suggestions, and prompt reflection to increase teachers’ confidence in implementing with fidelity (Raney & Robbins, 1989).

Although the AIR synthesis was useful for clarifying the role of coaches in supporting implementation by outlining the various forms and practices, there is still little that is known about teachers’ perceptions of the value or acceptability of these different coaching elements. Acceptability and perceived value are aspects of social validity; in general terms, social validity is considered to be society’s attribution of a product’s value (Wolf, 1978). While the intended outcomes need to be perceived to be of benefit or need, the processes through which the outcomes are achieved also need to be acceptable. If a teacher does not accept the need for an intervention, they are unlikely to engage with any aspect of the programme, including the coaching model. However, it is also possible that while teachers do perceive a need for the intended outcomes of a P&P programme, they do not consider the processes through which the intervention achieves its aims (for example, the coaching model) to be acceptable. The acceptability of the P&P programme and the attached coaching model in particular is pertinent, as a key function of the coach is often to ensure that the programme is implemented with the high level of fidelity required to achieve the intended outcomes, and so it is vital that teachers engage in coaching practices (Albin, Lucyshyn, Horner, & Flannery, 1996). Thus, it is important that the specific practices that teachers do find valuable are identified, in order to develop an acceptable coaching model; this will help to ensure that the mechanisms of change thought to be vital to the programme’s success are triggered.

Although the social validity of intervention processes has been highlighted in the implementation literature as a key area requiring attention (Miramontes, Marchant, Heath, & Fischer, 2011), there are few studies that have explored this in detail. In particular, the coaching aspect of P&P programmes lacks clear research into its acceptability, both in the interventions’ countries of origin and when imported into other countries. However, one study (Wehby et al., 2011) did explore the social validity of the coaching model within one school-based intervention, the GBG. It was found that a high-quality coaching relationship and a positive perception of the intervention’s social validity by teachers were significantly associated with fidelity, indicating that social validity and the quality of the coaching relationship may have a reciprocal role regarding teachers’ adherence to intervention components. This suggests that teachers’ responses to the coaching model are an important factor in determining an intervention’s implementation, and hence its ultimate success. However, the specific roles and practices that the coaches engaged in, and teachers’ responses to these, were not explored, and so the characteristics of the coaching relationship deemed to be most valuable to teachers are not known.

A greater understanding of the elements perceived to be most beneficial could enable the coaching model to be tailored, increasing its social validity and thus increasing the likelihood of achieving the levels of fidelity required to achieve the intended outcomes. Kretlow and Bartholomew (2010) noted in their review of coaching models that of the studies that did collect data on social validity, all reported that teachers rated coaching activities positively. However, these data were collected quantitatively, and so ascertaining the coaching practices that teachers engaged in, and those perceived to the most beneficial, was not possible. A further exploration utilising qualitative data may enhance understanding in this area, by providing greater insight into the elements of coaching considered to be the most valid to teachers.

With the increasing interest in early prevention in the UK, specifically regarding social and emotional learning (SEL) and mental health (Humphrey et al., 2016a), P&P programmes, including those containing a coaching element, have been imported to meet demand (e.g. PATHS, Humphrey et al., 2015; GBG, Coombes et al., 2016). However, although these programmes have been demonstrated to be effective in their country of origin, cultural transferability cannot be assumed (Wigelsworth et al., 2016). Indeed, in the UK, a number of studies that tested imported programmes known to be successful within their country of origin reported null or mixed intervention effects (Wigelsworth et al., 2016). For instance, findings have been mixed for PATHS within UK primary schools, despite its effectiveness in the USA (Greenberg & Kusché, 1993). While Little et al. (2012) reported null intervention effects, Curtis and Norgate (2007) reported significant improvements across all five dimensions of the Strengths and Difficulties Questionnaire. However, Humphrey et al. (2016a, b) noted that although there were reductions in teacher-reported emotional symptoms in children, this was the case for participants in both the intervention and usual practice conditions of the randomised controlled trial (RCT). Thus, there may be some factors influencing the successful implementation of these imported programmes.

As the testing and implementation of imported P&P programmes that include a coaching model becomes more commonplace in the UK, cultural transferability is a key factor that must be considered. Previous literature has indicated that cultural context is an important consideration when introducing a new intervention or practice within a new setting (Lyon et al., 2014; McKleroy et al., 2006); Castro, Barrera, and Martinez (2004a, b) observed in their programme adaptation framework that the key issue in the process of importing interventions is striking a balance between fidelity and cultural adaptability. This can pose a particular issue when importing school-based interventions given that education systems can differ greatly between countries, particularly where programmes require a high level of fidelity (Wigelsworth et al., 2016). Indeed, it is rare for imported programmes to be implemented exactly as intended, as local adaptations are common in order to improve the goodness-of-fit with the different education systems, cultural values and expectations of the adopters (Berkel, Mauricio, Schoenfelder, & Sandler, 2011; Ferrer-Wreder, Sundell, & Mansoory, 2012; Lendrum & Humphrey, 2012; Ogden & Fixsen, 2014). While such adaptations may be beneficial in terms of enhancing acceptability and sustaining longer-term implementation (Ferrer-Wreder et al., 2012; Lendrum & Wigelsworth, 2013; Lendrum & Humphrey, 2012), there is also evidence that the greater the number of modifications, the higher the risk that crucial components are lost, and so the required mechanisms of change are not triggered (Blakely et al., 1987). A concern regarding cultural transferability often focuses on sustaining implementation with sufficiently high levels of fidelity to achieve the intended outcomes (Ferrer-Wreder et al., 2012). As high levels of fidelity are associated with intervention effectiveness (Durlak & DuPre, 2008), programme developers often utilise coaches to ensure that implementers are adhering to the programme structure.

There are contentions between programme designers aiming for teachers to implement with high fidelity—to produce the intended outcomes, and teachers in UK schools needing to adapt an imported intervention and its implementation strategies to fit with their practices and contexts. While research indicates that fidelity and adaptability are not mutually exclusive, there is evidence to suggest that there is a critical balance that ought to be sought between the two (Lendrum & Humphrey, 2012). However, Castro et al. framework (2004a, b) posits that deep structural adaptations may be necessary when transferring an intervention across cultures. Thus, the role of the coach when supporting imported programmes may be crucial in maintaining high levels of fidelity, while also ensuring that any adaptations are in line with both the programmes’ underlying theory of change and the local culture. If the coaching model, or aspects of it, is not perceived by teachers to be acceptable or valuable, then it is unlikely that they will engage with the process, and thus fail to find the appropriate balance of fidelity and adaptation needed to support high levels of implementation.

In summary, cultural transferability and social validity are likely to play a role in teachers’ evaluations of their experiences with the coaching model of an imported intervention (Lendrum & Wigelsworth, 2013). However, there is little research on how teachers in UK classrooms perceive engagement with a coach, and whether the coaching practices undertaken are deemed acceptable or valuable. Additionally, previous studies have not explored whether coaching practices complement pedagogical approaches typically utilised by UK teachers. For instance, although research suggests that certain practices such as observations and feedback are associated with higher levels of fidelity (Kretlow & Bartholomew, 2010; Stormont et al., 2015), existing high-pressure inspections by regulatory boards such as Ofsted may mean that UK teachers perceive observations negatively (Blower, 2014; Illingworth, 2007). Furthermore, finding time to accommodate a visiting coach could be seen as a competing priority with the delivery of curriculum content (Stallard, 2013); indeed, research suggests that teachers in the UK are already struggling with the number of demands placed on them (Blower, 2014; Illingworth, 2007). Such cultural issues could affect the socially validity of the coaching model in the UK, which may impact on the likelihood of the programmes being implemented as intended. If implementation is low, then there is an increased likelihood that the key mechanisms of change will not be triggered, and the P&P programme will ultimately be unsuccessful. Therefore, knowing the elements of the coaching model that UK teachers perceive to be valuable may be crucial in supporting high fidelity, so that the intended outcomes are achieved.

## The Current Study

The coaching model of P&P programmes is a relatively new concept in UK schools, and cultural transferability cannot be assumed; it is therefore unclear whether the model is socially valid to UK teachers. The social validity of the coaching model can affect how well the programme is implemented, which in turn influences whether the intended mechanisms of change and subsequent successful outcomes are attained. The current study explored teachers’ experiences and perceptions of the coaching model attached to two universal, school-based P&P programmes, GBG and PATHS, in order to help inform the development of social valid coaching models for UK schools.

### GBG

The GBG is an interdependent group-contingency strategy (Lastrapes, 2013) originally developed by Barrish, Saunders, and Wolf (1969) to be used by teachers alongside the curriculum in primary schools. The GBG is a universal, highly structured behaviour management programme (Coombes et al., 2016) designed to create a positive learning environment (Chan et al., 2012). The GBG is prescriptive, with a detailed manual outlining the steps that teachers are expected to implement. Prior to implementation in the UK trial, the manual was “Anglicised” in terms of language, spelling and any cultural references before being given to teachers. Fidelity to the manual was outlined by Chan, Foxcroft, Smurthwaite, Coomes, & Allen (2012) in their logic model as one of the mechanisms required to produce change.

In addition to fidelity, coaching is considered to be the second key element of the GBG implementation model. The primary purpose of the coach is to support and encourage implementation; coaches typically observe a lesson, in which they complete a fidelity checklist, recording the aspects of the manual that were adhered to. They then provide direct feedback to the teacher, discussing or modelling areas for improvement, and highlighting areas of successful implementation. According to the logic model, coaches are required to visit teachers every 2–3 weeks for 90 min (Chan et al., 2012). It is thought that coaches provide the necessary support to foster the independence that is required to sustain long-term, high-quality implementation (Poduska & Kurki, 2014).

### PATHS

PATHS is a universal SEL curriculum, originally developed by Greenberg and Kusché (1993), designed for use in primary schools (CPPRG, 1999). PATHS aims to help children manage their behaviour, understand their emotions, and work well with others by targeting specific risk and protective factors. PATHS is a manualised intervention, including scripted lesson plans to help teachers achieve the high fidelity intended by programme developers (Humphrey et al., 2016b). In the UK trial of PATHS, the materials were “Anglicised” before they were provided to teachers, and teachers were also given an implementation guidance manual to emphasise the importance of effective delivery (Humphrey et al., 2016a).

In order to ensure optimal fidelity, technical support and assistance is provided to teachers by trained PATHS coaches. The coaches’ role is to support teachers with implementation by helping them deliver PATHS with fidelity, modelling lessons, providing feedback, and supporting teachers to generalise and integrate PATHS techniques into other aspects of teaching. Coaches are also thought to be the school liaison for PATHS (Barnardo’s, 2015; Berry et al., 2015). In the UK trial, coaches initially visited teachers for one lesson twice every half term, although this reduced over time and visits were arranged as often as required.

## Method

### Context of the Study

The current study utilised qualitative interview data collected as part of two cluster RCTs conducted to evaluate the effects of two universal, school-based, preventive interventions: the GBG and PATHS. As part of the trials, teachers received ongoing support from coaches to implement the respective interventions. There were three PATHS coaches (2 female, 1 male), all of whom had previously worked as teachers and were educated to Masters level. There were between four and six GBG coaches at any one time (nine in total, 3 males 6 females), although turnover was higher in the GBG trial. All GBG coaches had previously worked as teachers or education professionals. All teachers received an approximately equal number of visits from their coach. Coaches all received intensive initial training and ongoing supervision from the programme developers.

These trials were each conducted over 2 years by research teams at the University of Manchester (PATHS: 2012–2014; GBG: 2015–2017), and were amongst the first to evaluate these interventions in a UK context. The GBG trial focused on the impact of the intervention on pupils’ academic attainment and behaviour in Years 3 and 4 (ages 7–9). The PATHS trial focused on the social and emotional wellbeing of pupils in Years 3–6 (ages 7–11). Both trials included a parallel implementation and process evaluation. Both studies were approved by the University of Manchester Research Ethics Committee (GBG Reference: 15126; PATHS Reference: 11470).

### Participants and Procedure

The sample consisted of 33 Year 3 (pupil ages 7–8) and Year 4 (pupil ages 8–9) teachers in schools in Greater Manchester, the Midlands, and South and West Yorkshire, England. Qualitative interviews were conducted twice a year with all of the 14 GBG teachers from the six self-selecting case study schools involved in the wider trial. However, data were not collected from all teachers at each time point due to factors including teacher availability and attrition. All Key Stage 2 PATHS teachers were interviewed annually in the wider trial, although only interviews from 19 Year 3 and Year 4 teachers in six PATHS schools were selected for analysis at random from the larger dataset for the present study, due to the larger volume of data. Table 1 provides an overview of the participants involved in the present study. Participants’ fidelity scores collected as part of the wider trials are also included in Table 1 to provide some context for the findings. Participant’s fidelity scores were obtained through the use of a structured observation schedule, consisting of the steps outlined in the respective manuals. Fidelity was scored on a binary yes/no basis and summed for GBG, and was rated on a scale of 1–10 for PATHS. Percentage score ranging from 0 to 100% were then produced for both programmes.

**Table 1:** Details of participants involved in the study

Teacher	Year group	Number of interviews	Fidelity (%)
*GBG*			
GT1	3	2	64
GT2	3&4	4	65
GT3	3	1	–
GT4	4	2	68
GT5	4	2	41
GT6	3	2	76
GT7	3	1	61
GT8	3	1	52
GT9	3	2	77
GT10	3	2	68
GT11	4	2	68
GT12	4	2	75
GT13	4	1	–
GT14	3	2	77
*PATHS*			
PT1	3	1	45
PT2	3	1	70
PT3	3	1	100
PT4	3	1	–
PT5	3	1	–
PT6	4	1	85
PT7	4	1	95
PT8	3	1	95
PT9	4	1	75
PT10	3	1	55
PT11	3	1	80
PT12	4	1	55
PT13	4	1	80
PT14	3	1	100
PT15	4	1	65
PT16	3	1	100
PT17	4	1	75
PT18	3	1	80
PT19	3	1	75

### Interviews

Data were collected using semi-structured interviews, whereby a schedule acted as a guide to ensure specific topics were addressed, while also allowing for unanticipated responses to emerge (Galletta, 2013). Bespoke interview schedules to explore the key aspects of implementation were developed a priori for each trial. These included questions relating directly to teachers’ experiences and perceptions of the coaching models. Prompts and probes were utilised where necessary to encourage participants to elaborate on their answers and to clarify unclear responses. Interviews were conducted by members of the research team in a private room in the schools. Interviews lasted approximately 30 min.

### Analysis

**Fig. 1:**
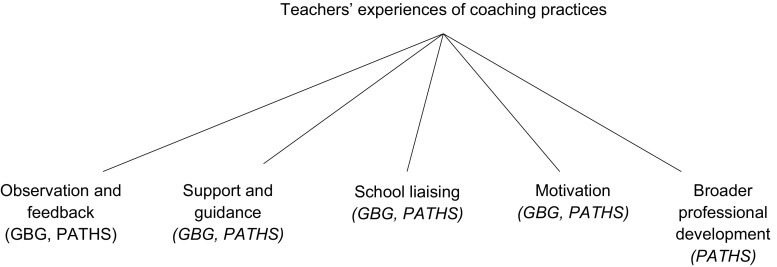
A priori thematic map

Transcripts were analysed jointly by the first two authors and so were divided equally by school prior to analysis. A hybrid thematic analysis at a semantic level was undertaken in accordance with Braun and Clarke’s six-phase guide (2006). Interviews were analysed in two stages, using NVivo to manage the process (https://www.qsrinternational.com/nvivo/). Analysis was conducted both inductively and deductively. The first stage involved developing a coding schedule utilising a priori themes that were informed by the extant literature to identify the practices that teachers reported engaging in with their coach. Figure 1 shows a thematic map of the a priori themes, with the global theme “teachers’ experiences of coaching practices” and six organising themes of specific practices, for example “observation and feedback”. The organising themes stemmed firstly from GBG and PATHS literature and were then developed with consideration of the wider coaching research, particularly drawing on AIR’s report (2005). Each transcript was then analysed following this coding schedule.

**Fig. 2:**
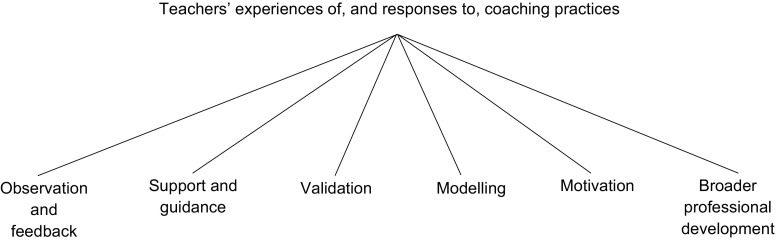
Thematic map of findings

Following the first stage of analysis, emergent themes were identified, and any additional codes were developed for these prior to the second stage, which focused on teachers’ responses to the practices previously identified. For this stage, the authors exchanged transcripts to allow a fresh perspective. The transcripts were then analysed again, utilising the amended coding schedule (see Fig. 2), incorporating both inductive and deductive themes. Exchanging transcripts meant that both authors analysed all transcripts, helping to ensure that coder biases were guarded against.

## Results

Figure 2 shows a thematic map of the themes identified in the data, including both a priori and emergent themes. Almost all of the a priori themes were evident within the data, with the exception of “school liaising”. Two basic themes emerged during analysis, “validation” and “motivation”; as such, six themes were identified. It is interesting to note that while almost all teachers made comments surrounding “observation and feedback”, there were discrepancies in the experiences of the other practices amongst the teachers of the two P&P programmes. For instance, “support and guidance” was predominantly identified by GBG teachers, although PATHS teachers also commented that this would have been valuable, while “modelling” was discussed mainly by PATHS teachers. Furthermore, although “broader professional development” was also discussed by teachers implementing both interventions, comments were relatively infrequent. This was to be expected regarding the GBG, as this practice is not incorporated into the model. However, supporting teachers to generalise PATHS techniques is defined as a key coaching practice in the PATHS model (Berry et al., 2015), and is also mentioned in coaching models of other P&P programmes (e.g. The Incredible Years; Reinke et al., 2012b).

### Observation and Feedback: “Someone Who’s Wanted to See the Game and Help”

Almost all teachers described being observed. Most GBG teachers specifically recalled that their coach used the fidelity checklist and many described feedback as being based on this, suggesting that their coach adopted a technical role. This feedback often followed a similar sequence: “going through the feedback of what she’d observed… we talked about why such a thing is good… what we could improve on next, what we’re working towards” (GT4). This pattern was not evident within PATHS teachers’ interviews, which typically included more general statements: “she gave us feedback on the lesson” (PT2).

Three teachers described initial apprehension about the observation process, which may be partly attributable to the frequent use of observations within the UK education system: “I was a bit worried… just thought ‘no not more observations’… but it is good” (GT6). Four teachers appeared relieved that observations appeared to focus on the intervention, rather than their practice: “it’s great to have someone… watching, not feeling like they’re judging or anything, and they’re just watching the game as opposed to watching us” (GT1). All commented that initial apprehensions about observations were unfounded and that the feedback was useful. One teacher discussed this at length, demonstrating a link with “validation”: “at first I was… a bit like ‘oh somebody else to come and watch me’ but… that’s not been the case… it’s just a helpful person who’s been really nice [it] boosts your confidence like ‘oh yeah I’m doing this right’ so it’s been good” (GT14).

Teachers described conversations as “very much dialogue” (GT13) with coaches aiming to encourage reflection: “I saw you do this, why did you do this? I’ve seen this, how could you make this work better?” (GT13). Teachers were generally positive about the “constructive feedback” (GT10) provided following observations, with 17 teachers commenting on the “really helpful process” (GT13): “it’s been brilliant because… you don’t look at your own work as critically as… somebody else would and… find the things that could possibly make it better…. Once you’ve delivered a lesson often, you don’t reflect as easily as someone… observing… it’s been really good” (GT11).

One teacher commented that, although initially helpful, the usefulness of observations and feedback declined over time:

“I think it’s really important. [Coach] has been fantastic. I do think we get observed a little bit too much… towards the end… we know what we can do with the game and our feedback was kind of the same all the time… but [coach] was really supportive in… everything that she did and ultimately she got us to that point.” (GT12).

### Support and Guidance: “You’re Not Just Being Left on Your Own”

Teachers described a range of experiences of support. Most of the GBG teachers were positive about the “really good advice” (GT14) and general support offered by coaches; “she’s just been really, really supportive” (GT5). However, not all PATHS teachers agreed with this: “I got the impression she was coming in just to see how the programme was going, not necessarily to support us” (PT3). Some PATHS teachers did comment that they were satisfied with the support they received, but only when prompted: “I’m happy with the level of support we’re getting at the moment” (PT5), while another reflected that “it’s probably about right but probably we should use it more than we do” (PT8).

A total of 11 teachers’ comments focused on fidelity, indicating that coaches were offering technical support: “you need people who actually know what they’re doing… to tell you, to show you how to do it” (GT8). This type of guidance was considered “really constructive, really positive… if you’re doing something wrong… she’ll point it out and give you a hand with it so it’s good, really good” (GT11). The coach was often regarded as a “safe pair of hands or an expert… to go to” (PT7) and several teachers found this type of guidance helpful due to the manualised nature of the intervention: “there’s a lot of things you get in a pack and there’s never a person to ask a question about. You can’t go ‘oh what does that really mean’ so it’s good to have someone” (PT9). However, others, particularly GBG teachers, felt that this was unnecessary as much of the knowledge offered by coaches was already present: “I don’t find it that useful… there’s nothing that I need to ask him about playing the actual game that I can’t figure out myself” (GT2). Indeed, one GBG teacher did not consider their coaches to be an expert: “I don’t think he knows any more about it than I do… I probably am more aware of how it works day to day than he is” (GT2). This may be due to the intervention being new to the UK, as another commented: “you could tell that it was very new…, there wasn’t necessarily loads of advice that [coach] could have given us at that time” (GT9).

Teachers reflected that the type of support that was useful varied across time. Initially, guidance on delivery was most useful: “at the beginning, my own lack of knowledge to do it [was a challenge] but… with the coach support… I’ve got into it quite easily and quite quickly” (GT11). Ongoing support via email appeared to be perceived as particularly beneficial to nine teachers: “she emails me… extra information on… the good things it could be used for really so yeah it’s been very helpful” (GT8).

General, rather than intervention-specific, support was also welcomed by four teachers: “sometimes it’s nice just to have someone to talk to, I’m not sure how helpful it is… it varies really, it just depends how stressed I am, just nice to get away from the classroom sometimes” (GT2). Linked to this, coaches’ broader knowledge and experience of education was also considered useful: “she’s got loads of experience herself from teaching so… it’s good because she can relate to what’s actually going on… her experience is brilliant… she’s been really supportive, she’s been great” (GT5).

### Validation: “It’s Reassuring to Know That You’re Doing the Right Thing”

This was an emergent theme consisting of two distinct but interlinked components; namely teachers’ experience of receiving praise, alongside reassurance that they were implementing the intervention as intended. Both elements were underpinned by teachers’ focus on fidelity.

Almost all teachers appeared to value positive feedback and guidance, with one teacher saying this was “the nicest thing about it” (PT4), particularly when it provided reassurance that they were delivering the intervention as intended: “it’s almost that reassurance that what you’re doing is actually what you’re supposed to be doing” (PT5). The validation that coaches provided was described by one newly qualified teacher (NQT) as particularly beneficial given their limited experience; “especially as a new teacher as well you can… think “oh am I doing it right? Am I doing it wrong?” but she always comes in with a positive and tells us how well we’re doing” (GT3). Another NQT commented that they would have preferred more of this: “a little more feedback maybe, only because I like to know if I’m doing well and ‘specially being an NQT just knowing that if I’m following it correctly that it’s okay” (PT1).

This is a particularly important theme as it not only highlights teachers’ perceptions that the key role of a coach is as a technical expert supporting fidelity and quality of delivery, but also indicates their beliefs that this may be best achieved through observations, with associated feedback delivered in a way that promotes confidence. Teachers recognised that the feedback they received was constructive, appearing to particularly appreciate that even “negative” feedback was framed in a supportive and positive way: “if you need to alter it a bit… she doesn’t say ‘oh you did that wrong’… she’ll nicely do it… and she’ll help suggest ways of improving it more… but she’s full of praise as well which is nice” (GT1).

### Modelling: “It was Really Good to Watch Someone Else Teach it”

Some PATHS teachers recalled that their coach had modelled a session and described this as helpful: “teaching a lesson as an example… I think all that support is brilliant” (PT9). However, teachers appeared to perceive the benefits of this differently; two teachers felt that having someone else deliver the lesson allowed them to observe pupils: “it was nice to see the children’s responses because sometimes when… delivering you don’t… get a chance to… see how everybody’s responding… that was really beneficial, to be able to see how PATHS is working from an outside kind of view” (PT6). However, another teacher valued the confirmation that they were delivering the intervention with fidelity, mirroring findings around “validation”: “I think the main reason I wanted to watch it was just to check I was doing it ok myself. So it was quite reassuring” (PT11); again, this suggests that teachers view their coach as a technical “expert”.

Although most PATHS teachers did not take advantage of the opportunity to observe their coach modelling a session, six commented that it would have been beneficial “to see like a PATHS person teach a lesson to see how you deliver it compared to how I deliver it” (PT2). Two GBG teachers commented that the modelling of a game during the initial training was valuable: “actually being able to see it being played… it was really easy to then model it in the classroom also… when the mentors modelled how to do it as well…, if I hadn’t have had that… I would have felt like a lot less confident in the classroom being able to do it” (GT9). This may have been sufficient to inform practice, as there was little reference to modelling in the GBG interviews. This may suggest that familiarity with the concepts and prescribed processes of delivery of an intervention may influence the extent to which modelling is considered to be a useful coaching practice.

### Motivation: “It also Reinvigorates You”

Teachers of both interventions reflected that the presence of a coach and the recurrent visits helped them to prioritise the intervention: “sometimes it’s a little bit easier to push to one side so having your mentor coming in again and again… brings it to a front a bit more” (GT9). This was often discussed in relation to the competing demands teachers felt they faced:

“because of the time constraints of fitting it in… it makes you do it… there’s a lot of pressures on us to do five million things, to fit so much into… the classroom… if we weren’t given that time and… knew that someone was coming into check up on you, it might go off by the wayside” (PT6).

Four teachers indicated that they found that the coaches’ visits acted as a helpful prompt and motivation to implement the intervention consistently, suggesting that the coaches’ role was technical: “[the observation] keeps people doing it basically… it also reinvigorates you when you come in because you’ve got someone watching you, you think ‘right better make it as make it as good as you can’” (PT7).

Although teachers commented that having ongoing visits “makes you do it” (PT6), this appeared to be more than a simple monitoring process. Instead, coaches helped teachers feel more engaged with the intervention and so more positive about implementing it: “quite often you forget but [coach] just… reminds us of what we’ve been talking about and keeps your morale up” (GT3). Teachers particularly valued this process as it encouraged them to prioritise the intervention, particularly during busy periods: “I thought it was brill… I think we need that just to keep us on track because… even though… I do really like it, the children love it… but especially when… teaching literacy and numeracy and coming up to assessments and stuff… it’s a little bit easier to push to one side” (GT9).

### Broader Professional Development: “It’s Just Nice to have Someone to Talk to About the Class”

Although teachers’ comments on this theme were relatively infrequent, they were present in several interviews across both interventions and typically took one of two forms. Two teachers reported that they received advice with generalising the intervention to wider practice, “I’ve had a few questions about how to use it outside of the… game” (GT6), while four highlighted that their coach provided guidance on aspects of practice that were separate to the intervention: “with the problems I’ve been having recently she was able to actually offer me some information on attachment theory… she’s just been really, really supportive, she’s been great” (GT5). Some teachers who did not receive this type of support commented that they would have found this valuable: “just to talk through some of the strategies that… have been put through the past… about how they could be modified and… perhaps… extended beyond the classroom” (PT3).

Teachers’ appreciation of the general guidance that coaches provided outside of the interventions suggests that they valued coaches’ experience as mentors or more knowledgeable peers rather than solely technical experts for a specific intervention: “I find it more… a supportive role for me as a teacher rather than the Good Behavior Game. It’s nice to just have someone to talk to about the class” (GT2). Some teachers described the benefit of receiving guidance with specific pupils, suggesting that they found their coach’s role to be that of a collaborator; the teacher who discussed receiving information on attachment theory commented “she was happy to send that to me and really help. She’s just been really, really supportive she’s been great” (GT5). Two teachers commented that although they did not have these particular issues, their coach’s insight would have been appreciated if this were the case: “if I had children with certain… behavioral difficulties then I think I would like to call on her more” (PT10).

## Discussion

The extant literature base currently lacks a clear universal definition of coaching, and in particular, there is a lack of agreement regarding the practices that coaches engage in with teachers (Becker, Bradshaw, Domitrovich, & Ialongo, 2013; Stormont et al., 2015). Indeed, even coaches often feel that their job role lacks a clear definition, and that this can create problems (Poglinco et al., 2003); for example, if there is mismatch between a teacher’s and a coach’s expectations. Furthermore, while coaching models often detail the intended practices of the coach, it is unclear whether teachers perceive these to be happening in their coaching visits. The present study therefore contributes to the literature in this area, by identifying the practices that coaches engage in from the perspectives of teachers, and which of these practices are perceived to be beneficial. The present study also looks at perceptions of coaching models specifically in a UK setting. The coaching model of P&P programmes is a relatively new concept in UK schools, and its cultural transferability cannot be assumed. This study therefore contributes to the evidence base in this area, by exploring the social validity of the model to UK teachers.

Four themes were identified in the present study that were consistent with existing literature regarding the practices that coaches most commonly engage in, namely “observation and feedback”, “support and guidance”, “modelling” and “broader professional development”. It appears that being observed and receiving subsequent feedback was a core element of coaching visits, with almost all teachers in both trials describing engaging in this process with their coach. This is frequently discussed as a key practice in other coaching literature (Reinke, Stormont, Herman, & Newcomer, 2014; Stormont & Reinke, 2012) and is unsurprising considering one of the primary purposes of the coaching model in both the GBG and PATHS is to monitor and sustain high levels of fidelity (Berry et al., 2015; Chan et al., 2012). Indeed, teachers’ focus on fidelity when describing the coaching practices were present across several themes, including “support and guidance”, and the emergent theme “validation”. Support from a coach is often cited as critical for promoting implementation fidelity (Reinke et al., 2014), and so teachers’ reports of this practice are consistent with the coaching literature.

It is noteworthy that while these practices are incorporated in both the GBG and PATHS coaching models (Becker et al., 2013; Berry et al., 2015; Reinke et al., 2012a), there were discrepancies in the experiences of them amongst the teachers, with the relative frequency of the comments differing between the two P&P programmes. However, it was not possible to ascertain why these discrepancies existed between the broader coaching literature and the findings from the present study; thus it is currently unclear whether coaches were unaware of the requirements to engage in these practices, or whether there were factors causing them to adapt the coaching model. For instance, coaches may have been responding to their own perceptions of the type of support specific teachers need. Previous research by Poglinco et al. (2003) into America’s “Choice Schools” suggests that both issues may be influencing coaches’ practices; they found that coaches all defined their roles differently, and that there was a great deal of uncertainty amongst coaches regarding their roles and responsibilities. Perhaps a further exploration could be conducted regarding coaches’ awareness of required practices, and their decisions to engage in them.

Two new themes emerged during the analysis in the present study: “validation” and “motivation”. Although these are not commonly referred to as key practices in the extant coaching literature, they are consistent with the reasoning behind other practices. For example, both teachers in the present study and the wider literature frequently refer to the key role of the coach in sustaining implementation over a long period of time (Han & Weiss, 2005; Kretlow & Bartholomew, 2010; Reinke et al., 2012b). Motivating teachers to continue implementation is therefore likely to be a practice that the coaches consider to be valuable to the success of the programme, particularly in UK schools where teachers already experience multiple competing demands being placed on them (Blower, 2014; Stallard, 2013). Conversely, resistance to coaching is often characterised by low levels of teacher motivation (Reinke et al., 2012b), and so coaches may be attempting to address this before it hinders teachers’ engagement with the intervention.

Several coaching practices, such as observations, emphasise the importance of implementing with adherence to the manual. It is probable that if teachers are made aware of the value placed on fidelity, but are new to the intervention and therefore lack self-efficacy in this area, that coaches will be providing frequent reassurance, and that teachers will be placing an increased weight on the presence of this practice. Indeed, research suggests that providing teachers with a coach leads to reports of greater self-efficacy and the ability to maintain newly learned practices (Forman, Olin, Hoagwood, Crowe, & Saka, 2009; Stormont & Reinke, 2012).

In addition to identifying the coaching practices that teachers perceived their coach to be engaging in, the present study also aimed to explore teachers’ responses to these practices, to assess the social validity and cultural transferability of the coaching model of P&P programmes in the UK (Stormont et al., 2015). Evidence of an association between the social validity of an intervention, and the degree to which it is implemented with high fidelity (Wehby et al., 2011) demonstrates the need for intervention developers to establish a model that is acceptable. However, while the social validity of coaching models has been established in their countries of origin (Stormont et al., 2015), it is also vital that it is assessed when importing programmes, as the acceptability and perceived value of an intervention can vary between cultures (Lendrum & Wigelsworth, 2013). The practices that teachers reported as acceptable can be incorporated by intervention designers when developing and refining coaching models in the future, to increase the likelihood of imported P&P programmes being accepted and implemented as intended by teachers, to achieve the intended outcomes.

Teachers were generally positive regarding the coaching practices identified in the present study. In particular, teachers identified “observation and feedback” as a helpful process, despite initial reservations. There is a strong school inspection culture in the UK, with observations conducted by regulatory boards such as Ofsted to monitor teachers. This is a growing problem in the UK, with nearly a third of teachers leaving the profession within 5 years of qualifying (Education Committee, 2017), many citing the pressure of observations as a factor affecting this decision (Blower, 2014). Indeed, 80% of teachers are anxious about Ofsted inspections, and the same number reported that the increased frequency of observations contributed significantly to work-related stress (Illingworth, 2007). Thus, it was not surprising that teachers were initially apprehensive about additional observations. However, ultimately teachers viewed these observations favourably, and comments were often linked to other themes including “support and guidance”, “validation” and “modelling”. A key factor appeared to be the way in which feedback was provided following an observation, with teachers valuing feedback that was both constructive and framed positively, along with the reassurance that they were implementing the P&P programme well. Teachers also appreciated that coaches were not judging them as teachers, but were there to observe the intervention; this again could be due to teachers’ previously negative experiences of more personal observations.

Therefore, it appears that the way in which observations and feedback are conducted with UK teachers may be vital to the acceptability of the coaching model of these imported P&P programmes. Although some previous literature on coaching models suggests that coaches should be careful regarding the manner in which they provide feedback (Raney & Robbins, 1989), the importance of validation to teachers was a new finding in the present study. The knowledge that one of the most frequently cited benefits of coaches by teachers is the provision of praise and the reassurance that they are doing well can be utilised to enhance the acceptability of coaching models in the UK.

Teachers also appeared to value support and validation specifically due to the emphasis placed on fidelity in the two interventions in this study. In particular, the coaches’ role as a technical expert eased teachers’ concerns regarding implementing the interventions as intended. This finding is similar to previous research in the area, which suggests that teachers’ confidence in their coaches’ ability is associated with higher levels of fidelity. Interestingly, this confidence is also related to the overall social validity of the intervention (Wehby et al., 2011), and so it appears that utilising a highly skilled or experienced coach could not only increase the acceptability of the coaching model, helping to ensure that the intervention is implemented as intended, but also increase the likelihood that the overall intervention is acceptable to the teachers, and thus sustainable (Miramontes et al., 2011).

However, the support and reassurance surrounding fidelity may be particularly paramount in the present study due to the interventions being highly manualised and unfamiliar to the teachers. These experiences may change over time once teachers’ confidence develops. Indeed, some teachers did articulate concerns regarding the frequency of visits and the utility of the feedback towards the end of the trial, and similar issues were expressed in the UK pilot of the GBG (Chan et al., 2012). There is also evidence to suggest that teachers with fewer visits from their coach are less emotionally exhausted and report a more positive experience (Pas et al., 2015), and so balancing the frequency of coaching visits, particularly as implementation progresses, may increase the social validity of the model.

It was also noteworthy that teachers valued the coach acting as a motivator, reminding them to continue implementing the P&P programmes consistently, despite some concerns about the frequency of visits. They also made comments around the general support provided to them as a teacher under “broader professional development”. Teachers in the UK face increasing demands and competing priorities in the classroom, with pressure placed on them to adhere to the curriculum, achieve Ofsted’s priorities, and for pupils’ attainment to meet national targets (Blower, 2014; Illingworth, 2007). Indeed, 96.5% of UK teachers say that their workload is problematic (Blower, 2014). It would therefore be unsurprising if teachers were resistant to additional demands regarding the implementation of a P&P programme, and incorporating regular coaching visits. However, this was not always the case, and while teachers acknowledged the time constraints of fitting the programmes in, they appeared to welcome the coaching visits as an encouragement to implement them with the required frequency, and to have somebody there to talk to about their problems with the class. This therefore appears to be a valuable aspect of the coaching model, although it has not often been discussed in previous literature. However, it may be important that this practice is incorporated into GBG and PATHS coaching models, particularly in UK schools, in order to ensure that implementation is sustained over time.

The findings from this study provide several contributions to the evidence base in this area, and offer recommendations to intervention developers as to the ways in which the acceptability of the coaching model of P&P programmes can be enhanced when exporting them to the UK. However, this study also has several limitations that should be noted. Firstly, only teachers implementing two P&P programmes were involved, GBG and PATHS; while these programmes differ in terms of their design, with one being curriculum-based, and the other a behaviour management strategy, they are similar in terms of their highly structured, prescriptive format. Both have an emphasis on fidelity, which may be why practices relating to fidelity featured so highly in the findings, and appeared to be particularly valuable to teachers. Hence, these findings may not be representative of different types of P&P programmes in schools. Furthermore, as the PATHS dataset was substantially larger than the GBG dataset, it was unfeasible to utilise all interviews; therefore, potentially different experiences from other teachers may have been lost. Due to the present study utilising data taken from wider RCTs, only a small portion of the interview schedules were devoted to questions on the coaching model, and questions were relatively open-ended. It was therefore not possible to establish why some themes were mentioned relatively infrequently; whether it was due to coaches not engaging in those practices, or teachers not identifying or placing value on them. Further research could be conducted utilising more in-depth and focused questions regarding the coaching model with teachers. Finally, coaches’ fidelity was not explicitly monitored during the study. Although all of the coaches were trained by the respective programme developers prior to implementation, and then received ongoing supervision from the intervention developers and the head coaches (which was, in part, designed to ensure that the coaches were implementing the intended practices), the coaches’ guidelines provided by the intervention developers are generic, and, to the authors’ knowledge, no empirically validated model exists regarding coaching practices. There is also no evidence available to suggest that coaches’ fidelity to certain practices influences intervention outcomes and thus no way of monitoring their fidelity is available.
